# Low Level Constraints on Dynamic Contour Path Integration

**DOI:** 10.1371/journal.pone.0098268

**Published:** 2014-06-16

**Authors:** Sophie Hall, Patrick Bourke, Kun Guo

**Affiliations:** School of Psychology, University of Lincoln, Lincoln, United Kingdom; University of Groningen, Netherlands

## Abstract

Contour integration is a fundamental visual process. The constraints on integrating discrete contour elements and the associated neural mechanisms have typically been investigated using static contour paths. However, in our dynamic natural environment objects and scenes vary over space and time. With the aim of investigating the parameters affecting spatiotemporal contour path integration, we measured human contrast detection performance of a briefly presented foveal target embedded in dynamic collinear stimulus sequences (comprising five short ‘predictor’ bars appearing consecutively towards the fovea, followed by the ‘target’ bar) in four experiments. The data showed that participants' target detection performance was relatively unchanged when individual contour elements were separated by up to 2° spatial gap or 200 ms temporal gap. Randomising the luminance contrast or colour of the predictors, on the other hand, had similar detrimental effect on grouping dynamic contour path and subsequent target detection performance. Randomising the orientation of the predictors reduced target detection performance greater than introducing misalignment relative to the contour path. The results suggest that the visual system integrates dynamic path elements to bias target detection even when the continuity of path is disrupted in terms of spatial (2°), temporal (200 ms), colour (over 10 colours) and luminance (−25% to 25%) information. We discuss how the findings can be largely reconciled within the functioning of V1 horizontal connections.

## Introduction

A fundamental process of human visual perception is contour integration, whereby discrete contour elements are integrated into coherent global (whole) shapes. This contour integration serves important visual functions, such as boundary identification and figure-ground segregation [1]. For static visual stimuli, contour integration is a well-established research topic in visual psychophysics and neuroscience. However, we know relatively less about how spatially and temporally separated dynamic features are processed in contour integration tasks.

The well-documented literature on the spatial constraints of contour integration has demonstrated the crucial role of relative spacing, angle and axial offset between static neighbouring contour elements in the grouping process [2]–[5]. Increasing the spacing between adjacent features reduces contour detectability. Observers' performance drops to chance levels when the contour spacing is beyond a critical range, about 2° between collinear line segments [6] or 10λ between collinear Gabor patches [5], [7]. Introducing misalignment or orientation jitter relative to the contour path also effectively decreases contour detectability [2], [5]. Observers even show difficulty in grouping two line segments when they are misaligned by as little as 1′ [8]. Variability in the colour and luminance of the contour elements can further affect integration performance [9]. Our visual system is biased to group elements of the same colour [10] and also shows better detection performance to luminance-defined (achromatic) contours [11]–[13]. These stimulus parameters (e.g., spatial, temporal, alignment and luminance features) influence neuronal contextual modulation in primary visual cortex (area V1) [14], [15]. Neurophysiologically, it has been proposed that V1 neurons play a fundamental role in contour integration, possibly via intrinsic long-range horizontal connections that link neurons with similar orientation preferences but non-overlapping receptive fields (RFs) [3], [16] and/or feedback projections from higher visual areas that process more sophisticated information (such as colour) or information from more extensive portions of the visual field by virtue of their large RFs [12], [17].

Less is known about the temporal constraints of contour integration. The majority of studies investigating this topic have focussed on the importance of temporal synchrony [1], [8], [18]–[20]. In typical collinear flanker-target-flanker design, the flanker facilitation has the maximum effect when the target precedes the flanker by 20–80 ms and has no effect when the target-flank separation is longer than 150 ms [20]–[22]. Studies have also shown that global contour integration does not demonstrate strong dependency on the temporal frequency of Gabor patches [19] suggesting it is a rapid process that is likely to involve fast horizontal connections in V1 [6], [23].

**Figure 1:**
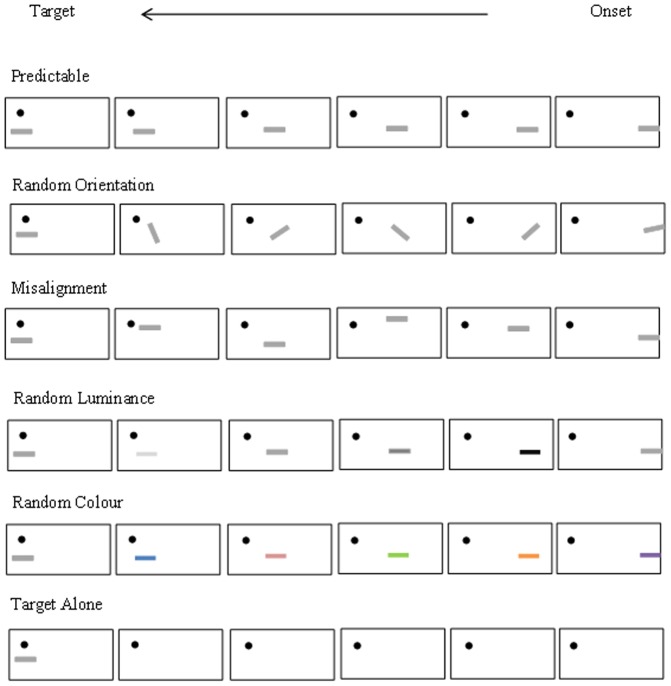
A demonstration of the stimuli conditions. Non-scaled demonstration of the stimuli conditions used in the experiments.

In the natural visual world, objects and scenes around us often occur and move in statistically predictable ways to create a stream of visual inputs which are spatially and temporally coherent [24], [25], such as the trajectory of a car moving on the motorway. Through evolution and development, our visual system should be able to effectively group relevant information across space and time, and exploit this spatiotemporal regularity when processing current visual inputs. This hypothesis has been tested by a few empirical studies using simplified dynamic visual stimuli to mimic natural spatiotemporal regularity [26], [27]. In our previous studies we presented human observers with a dynamic stimulus sequence comprising four collinear short bars (predictors) appearing consecutively towards the fovea followed by a target bar at fixation (see Fig. 1 for an example). Our paradigm combines the principles of two well-established psychophysical paradigms: the flanker facilitation task [3] and the contour integration task [2]. The flanker facilitation task typically requires the participant to make a brightness judgement, or target present/absent judgement on a central line segment that is flanked by two lines on either side of the target. Reports show that optimally positioned (co-linear) flankers increase target detection in comparison to target alone presentation and orthogonally positioned flankers [3]. The contour integration task often requires participants to make a 2-interval forced choice decision (target present/absent) on whether arrays of oriented elements (e.g., Gabor patches) contain a set of elements which are aligned to form a path. Reports show that observers can detect paths with relatively large element spacing, but reducing the alignment of the path elements significantly reduces path detection [2]. Our paradigm involves both contour integration and target detection or judgement tasks. However, whereas these classical paradigms requires the observer to detect a static central target or static curved contour path from distractor elements, our task needs the observer to integrate the dynamically presented, straight contour path elements in the absence of distractors.

Using this paradigm our studies show that observers' orientation judgment of the target bar was biased towards the orientation of the predictors [24]. This bias was much stronger for the predictors presented in a highly ordered and predictable sequence than in a randomised order. Participants also needed less contrast and showed quicker reaction times to detect the foveal target embedded in this predictable spatiotemporal stimulus structure, than in a randomised predictor-target sequence or presented in isolation without any predictors [25]. Clearly, these spatially and temporally separated collinear predictor bars were efficiently integrated as a coherent spatiotemporal contour path. Recordings of single-neuron responses in rhesus monkeys [28] and event-related potentials (ERPs) in humans [29]–[31] suggest that V1 neurons may be involved in this dynamic contour path integration, but this still remains unclear.

In the current set of studies, we aimed to further investigate the parameters under which the visual system could group dynamic contour elements to modulate performance in a target detection task. Human contrast detection performance of a briefly presented foveal target bar embedded in a dynamic contour path (typically comprising six short collinear bars appearing consecutively towards the fovea) was examined. The spatial and temporal interval between neighbouring bars, the colour, luminance, orientation and alignment of individual bars in the dynamic sequence were systematically manipulated in four separate experiments. We report each experiment separately in the results section, and include a brief rationale and method section before reporting the results in individual experiment. Our findings illustrate that dynamic contour path integration shows little sensitivity to disruption in spatial, temporal, colour, luminance and alignment information, but is perhaps more influenced by orientation cues.

## Methods

The methods outlined in this section describe the general protocol and design employed across the four experiments; the individual experimental details are outlined in their respective sections in the results section.

### Ethics Statement

Informed written consent was obtained from each participant, and all procedures complied with British Psychological Society “Code of Ethics and Conduct”, and with the World Medical Association Helsinki Declaration as revised in October 2008. The ethical committee in the School of Psychology, University of Lincoln approved the study.

### Participants

In total 16 participants (including two authors), aged between 18- and 43-years (22 years ±6, Mean ± SD), took part in this study. There were 9 females. All participants had normal or corrected-to-normal visual acuity.

### Design

Visual stimuli were presented through a ViSaGe Graphics system (Cambridge Research Systems, UK) and displayed on a non-interlaced gamma-corrected colour monitor (100 Hz frame rate, 40 cd/m^2^ background luminance, 1024×768 pixels resolution, Mitsubishi Diamond Pro 2070SB). At a viewing distance of 57 cm the monitor subtended a visual angle of 40×30°. The visual stimuli comprised six short bars (1° length, 0.1° width) appearing successively towards the fovea following a collinear path (predictor-target sequence, see Fig. 1 for examples), so that they created an apparent motion stream towards the presentation of the target bar. Unless specified in individual experiments, the first five ‘predictor’ bars with 15% contrast were presented in the right peripheral visual field (the centre of the furthest predictor bar was 5° away from the fovea). The sixth ‘target’ bar was presented 1° below a small red fixation point (FP, 0.2° diameter, 10 cd/m^2^) in varying contrast (0%, 0.25%, 0.5%, 0.75%, 1%, 1.25%, 1.5%, 1.75%, 2%, 2.5%, 15%). Each bar was presented for 200 ms. Typically there was no spatial and temporal gap (or spacing) between adjacent bars. The bars were flashed in turn, in a position immediately adjacent (end-to-end) and in a time immediately preceding the next bar at successive positions. The location, orientation, luminance and colour of individual predictor bar were manipulated independently in different predictor-target sequences (the detailed manipulation of stimulus structure is described below for the individual experiments). Regardless of experimental manipulation of the predictor-target sequences, the horizontal target bar (1° length, 0.1° width) was identical and always presented 1° below the FP.

To familiarise the participants with the task a training session (normally 20 trials) was given before the formal test. During the experiments, the participants sat in a quiet, darkened room, and viewed the display binocularly with the support of a chin-rest. In each experiment four predictor-target sequences and 11 target contrasts were presented in a random order; so that neither the sequence nor the target contrast were predictable based upon the stimulus previously viewed. The trial was started by a 350 Hz warning tone lasting 150 ms followed by a delay of 1000 ms. A predictor-target sequence was then presented. Across four experiments we used ten conditions (target alone, predictable, 100 ms predictor gap, 200 ms predictor gap, 1° predictor gap, 2° predictor gap, random colour, random luminance, random orientation and misalignment; details are presented in the respective experimental sections). In each experiment four of these conditions were presented. The target alone and predictable sequence were displayed in all of the experiments. In the target alone condition no predictors were presented, only the target bar. In the predictable sequence five collinear predictors appeared successively towards the fovea in highly predictable spatial and temporal order, followed by the target; there was no spatial and temporal gap between adjacent bars (i.e., predictor 1→predictor 2→predictor 3→predictor 4→predictor 5→target). Two other conditions were selected based upon the aim of the experiment. The participants were instructed to maintain fixation of the FP throughout the trial, and to indicate, by pressing the ‘enter’ key on a computer keyboard as quick as possible, when they were reasonably confident that the target had been presented below the FP within this predictor-target sequence (target present/absent detection). No feedback was given. The inter-trial interval was set to 1500 ms. A minimum of 20 trials were presented for each target contrast, for each predictor-target sequence. During the experiments the observers were encouraged to have a short break if it was necessary.

**Figure 2:**
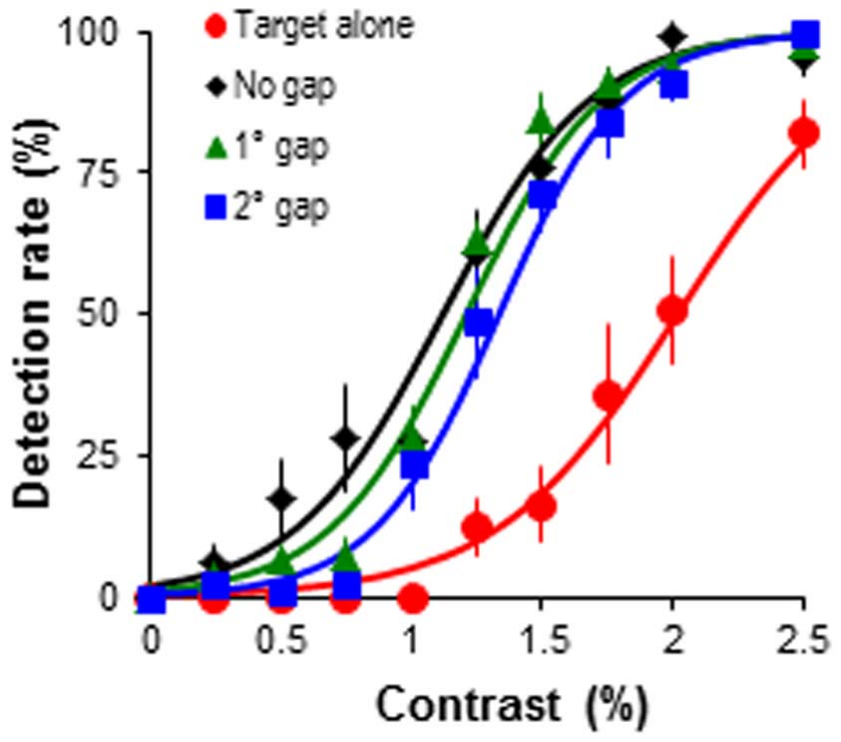
Target detection performance across spatial gaps. Target detection rate as the function of target contrast. The target was embedded in predictable predictor-target sequence, but the spatial gap (0°, 1°, 2°) between the adjacent bars was systematically varied. Error bars represent the standard error of mean.

**Figure 3:**
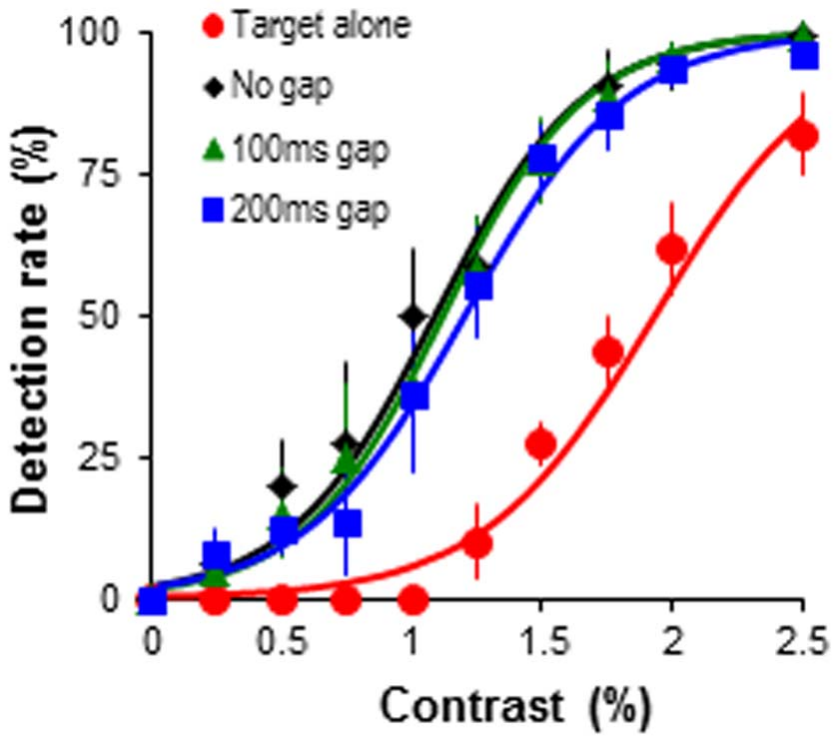
Target detection performance across temporal gaps. Target detection rate as the function of target contrast. The target was embedded in predictable predictor-target sequence, but spatial gap (0 ms, 100 ms, 200 ms) between the adjacent bars was systematically varied. Error bars represent the standard error of mean.

**Figure 4:**
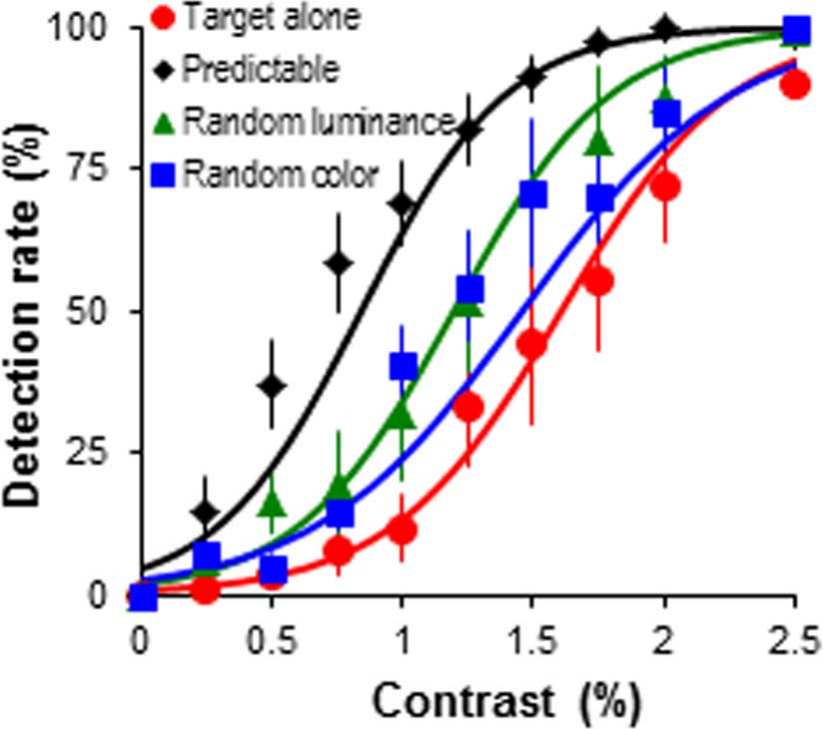
Target detection performance with randomised colour and luminance of the contour elements. Target detection rate as the function of target contrast. The target was embedded in predictable predictor-target sequence, but the colour and contrast of the adjacent bars was systematically varied (see methods). Error bars represent the standard error of mean.

**Figure 5:**
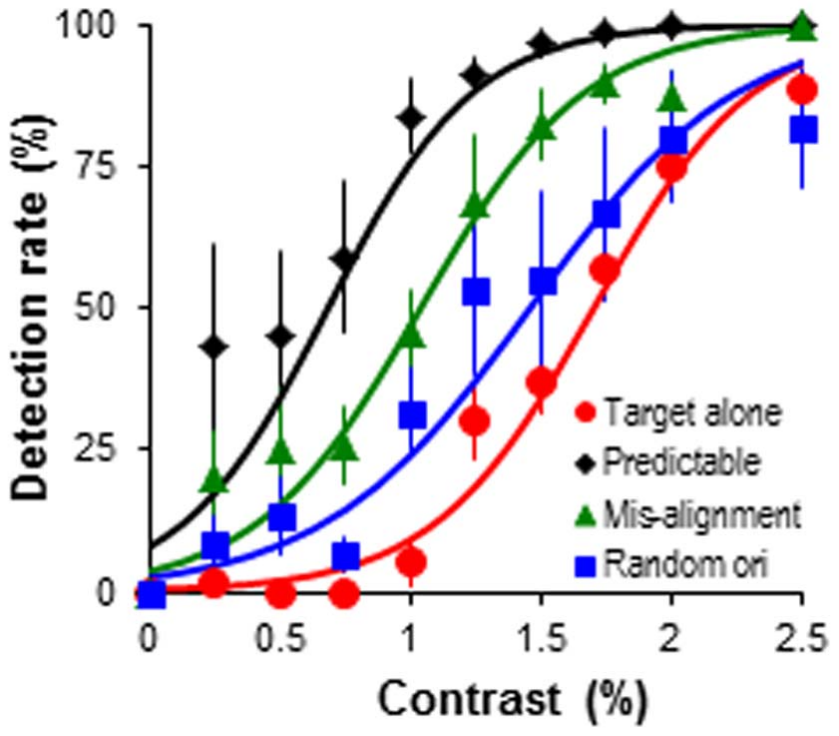
Target detection performance with random orientation and random alignment of the contour elements. Target detection rate as the function of target contrast. The target was embedded in predictable predictor-target sequence, but the alignment and orientation between adjacent bars was systematically varied (see methods). Error bars represent the standard error of mean.

The participants' detection performance (percentage of target detection judgment) was measured as a function of target contrast. Catch trials (0% and 15% target contrast) were used to correct for guessing target detection. Across the participants and predictor-target sequences, the mean hit rate for the presence of 15% target contrast was 99.6%±2.2, and the mean false alarm rate for the presence of 0% target contrast was 4.3%±6.8. Analysis was conducted on the data (detection rate) calculated after a bias correction. The detection rate for target presence with a tested contrast was calculated as (observed hit rate – false alarm rate)/(1-false alarm rate)×100 [32]. The normalised detection rates were plotted against the target contrasts and fitted with logistic psychometric functions (Fig. 2, 3, 4 and 5). Pilot testing showed that analysis Point of Subjective Equality (PSE) values did not accurately capture observer's sensitivity to the different parameter manipulations. By including data from all contrast points we can gain more information from the data than we can if we restrict our analysis to PSE values. Furthermore, in using the psychometric fitting we recognise that the fit to the data is not always ideal; therefore, it is more reliable to base the statistics on actual observed data as opposed to predicted fitted data. Therefore, target detection performance in different predictor-target conditions was analysed using repeated measures analyses of variance (ANOVA), with condition (stimulus sequence) and contrast (0–2.5%) entered as the within subjects factor in the initial analysis. Tukeys error adjustments were applied in pairwise comparisons and these are used to report the effects of condition across contrasts. Across all analyses conducted there was a significant main effect of contrast (*Fs*≥67.70, *ps* <.001, *η_p_^2^*≥93) illustrating better target detection performance the higher the target contrast (as would be expected). We do not report this main effect but instead focus on the main effect of condition and the interaction between condition and contrast. All interactions condition×contrast effects were analysed by comparing the effect of condition separately at each contrast point to avoid multiple post-hoc testing

## Experiments and Results

### Experiment 1: manipulation of spatial gap between predictors

With similar stimulus arrangements, Hall et al. [25] demonstrated that our detection of low-contrast targets depends heavily on the context of the predictor path. In comparison with the target alone sequence, participants showed increased detection rate and shortened reaction time in response to the target embedded in a predictable collinear predictor-target sequence (Fig. 1 in [25]). Such enhanced target detection performance could not be fully accounted for by response bias and uncertainty reduction (e.g., predictor presentation can reduce spatial and/or temporal uncertainty about target presentation), suggesting our visual system takes the regularity of the spatiotemporal contour path into account when interpreting incoming target information [24], [25]. In other words, spatially and temporally separated predictor information can be effectively integrated to facilitate the detection of the target.

The spatial and temporal parameters of contour integration are typically investigated by focussing on the role of relatively local mechanisms, such as flanker facilitation (as opposed to a more global integration process). For instance, the well-studied phenomenon of flanker facilitation or collinear facilitation has demonstrated that our contrast sensitivity to a low-contrast Gabor target is enhanced when presented in the context of spatially separated collinear flankers (flanker-target-flanker) [24], [33]. Studies regarding the spatial and temporal determinants of such flanker facilitation have further revealed that the amount of facilitation varies with the spatial and/or temporal gaps between the target and flankers. Spatially, facilitation is the greatest when the spatial gap between the target and the flanker is about 3λ or 4λ. Increasing or decreasing the spatial gap from this optimal distance leads to a significant reduction in the facilitation effect [33]–[35]. The effects of temporal gaps are not so cohesive. The majority of evidence suggests a short range determinacy on temporal integration, with flanker facilitation having maximum effect when the target precedes the flanker by 20–80 ms, and no effect when the target-flank separation is longer than 150 ms [21], [22]. However, it has also been reported that memory processes can be evoked in visual integration, with contrast detection performance facilitated when collinear flanker and target are separated up to 16 seconds [36]. Although there is considerable debate about the neural mechanisms underlying the flanker facilitation, the long-range horizontal connections in primary visual cortex seem to play a dominant role [16], [37].

To extend our knowledge of the spatial and temporal determinants of visual integration to dynamic contours (as opposed to flanker-target-flanker integration), we manipulated the spatial (experiment 1) and temporal (experiment 2) intervals between the presentations of adjacent predictor (contour) bars. We predicted that if the visual system could integrate contour information over different spatial and temporal intervals then the information provided by the predictor bars (the contour) would be used to bias target detection performance (i.e., target detection performance would be comparable across different spatial and temporal intervals between the bars). If dynamic contour integration is disrupted by spatial and temporal gaps the visual system will be less efficient at using the contour information to predict the target appearance and therefore target detection rates will decrease with greater spatial and/or temporal gaps.

#### Method

To examine to what degree spatially separating individual bars in the predictor-target sequence affect target detection performance, the stimulus structure was manipulated in four conditions: (1) *Predictable sequence:* see main methods, (2) *1*° *spatial gap:* the dynamic stimulus structure (including the number of the bars, the length and width of the bars) was the same to that in the predictable sequence, only the spatial gap between adjacent bars was increased to 1°; (3) *2*° *spatial gap:* the same as in condition 2, only the spatial gap between the adjacent bars was increased to 2°; (4) *Target alone sequence*: see main methods. Five volunteers participated in this experiment.

#### Results and Discussion

A 4 (stimulus sequences)×10 (target contrast levels) ANOVA revealed that compared to target alone sequence, predictable sequence significantly increased target detection rate (*F*(3, 12) = 20.80, *p*<.001, *η_p_^2^* = .84; Fig. 2) even with the large 2° spatial gaps (pairwise comparisons: all *ps*<.02). A significant condition×contrast interaction (*F*(27, 108) = 7.21, *p*<.001, *η_p_^2^* = .64) was analysed further. The results showed that the enhancement in contrast detection performance was most evident when the target contrast was varied 1–2.5% (*Fs*≥6.07, *ps*<.05). Unlike those results reported in flanker facilitation, the distance of spatial interval had negligible effects on the amount of facilitation in target detection performance. That is, increasing spatial gap between the predictors had limited effect of decreasing detection rate to target detection. Specifically, compared to the predictor-target sequence with larger spatial gaps (≥1°), only the detection rate for 0.75% contrast target was higher in the predictable sequence without spatial gap (*F* = 6.70, *p*<.01). For lower target contrasts (0.25–0.5%) no significant effects of condition were observed (*Fs*≤5.01, *ps*≥.05). For higher contrast targets (≥1%), the detection rate was indistinguishable among stimulus sequences with different spatial gaps (all *ps*>.05). This indicates that dynamic global contour path integration operates over relatively large spatial intervals.

### Experiment 2: manipulation of temporal gap between predictors

#### Method

To examine to what degree temporally separating individual bars in the predictor-target sequence affect target detection performance, the stimulus structure was manipulated in four stimulus conditions: (1) *Predictable sequence*; (2) *100 ms temporal gap:* the dynamic stimulus structure was similar as in the predictable condition, only the temporal gap between adjacent bars was increased to 100 ms; (3) *200 ms temporal gap*: the same as in condition 2, only the temporal gap between the adjacent bars was increased to 200 ms; (4) *Target alone sequence*. Five volunteers participated in this experiment.

#### Results and Discussion

A 4 (stimulus sequences)×10 (target contrast levels) ANOVA revealed that compared to target alone sequence, predictable sequence significantly increased target detection rate (*F*(3, 12) = 23.45, *p*<.001, *η_p_^2^* = .85; Fig. 3) even with the long 200 ms temporal intervals (pairwise comparisons: all *ps*<.02). Varying temporal interval between the presentation of adjacent bars (offset-onset delay as 0 ms, 100 ms, and 200 ms), on the other hand, had no clear effect on the target detection performance (all *ps*>.05). A significant condition×contrast interaction (*F*(27, 108) = 4.45, *p*<.001, *η_p_^2^* = .53) showed that the enhancement of the predictable sequence (with varying temporal intervals) was most noticeable between target contrasts 1–2.5% (*Fs*≥5.42, *ps*<.05). At contrasts below this (0.25–0.75%) no significant effects were observed (*Fs*≤2.82, *ps*≥.05). Across the tested target contrasts detection rates were indistinguishable when the temporal interval was varied between 0 and 200 ms (*ps*>.05).

Overall, experiments 1 and 2 revealed a robust facilitation effect of dynamic contour path on target detection. Disrupting this contour integration by increasing spatial interval up to 2° or temporal interval up to 200 ms between adjacent contour elements had very limited detrimental effect on target detection performance, suggesting that our visual system can integrate spatially or temporally separated events into a coherent representation when these events change according to a predictable temporal structure (pattern of changes over time). These results contribute to the debate in the literature on the spatial and temporal determinants of contour integration by supporting studies which suggest integration can occur over large distances [36] and we extend this to show this is true for dynamic global integration mechanisms as well as flanker-target-flanker integration mechanisms.

### Experiment 3: manipulation of predictor's luminance and colour

Horizontal connections in area V1 tend to connect neurons sharing the same orientation and colour preferences [38], [39], and V1 neurons which are sensitive to chromaticity show less sensitivity to orientation [40]. These neurophysiological studies have indicated that contour elements with the same luminance contrast or colour would be easier to integrate. Psychophysical studies have observed that we are biased to group static elements of the same colour [10]. Detection performance is slightly better to luminance-defined (achromatic) than colour-defined (chromatic) contours, with evidence suggesting that higher cortical areas are involved in processing colour-defined contours compared to luminance defined contours [11]–[13]. For instance, the integration of achromatic contour elements is approximately 100 ms faster than for chromatic contour elements [11]. Additionally, we can integrate achromatic contours efficiently over 4.6° element spacing, but this declines to 3.6° for blue-yellow contours and 2.9° for red-green contours [12]. However, it has also been reported that collinear facilitation for static targets is similar for chromatic and achromatic stimuli [41] and that the processing of both is likely to be sub-served by neurons in V1 and V2 [42]. As such there remains confusion as to the capabilities of early visual neurons in integrating chromatic and achromatic stimuli. To our knowledge, the effects of chromaticity have yet to be investigated with dynamic contour integration.

#### Method

The stimulus structure was manipulated in four sequences: (1) *Predictable sequence*; (2) *Random luminance sequence*: the dynamic stimulus structure was similar as in the predictable sequence, but the individual grey predictor's luminance contrast was randomly varied between −25% and 25% with 5% step (0% contrast was excluded); (3) *Random colour sequence*: the dynamic stimulus structure was similar as in the predictable sequence, the individual predictor's contrast was kept as 15%, but its' colour was randomly varied between 10 different colours (CIE 1931 colour space, x1 = 0.284, y1 = 0.597; x2 = 0.383, y2 = 0.513; x3 = 0.47, y3 = 0.449; x4 = 0.289, y4 = 0.213; x5 = 0.237, y5 = 0.223; x6 = 0.195, y6 = 0.234; x7 = 0.311, y7 = 0.353; x8 = 0.373, y8 = 0.424; x9 = 0.329, y9 = 0.292; x10 = 0.311, y10 = 0.328); (4) *Target alone sequence*. Six volunteers participated in this experiment.

#### Results and Discussion

A 4 (stimulus sequences)×10 (target contrast levels) ANOVA showed that randomising element colour and luminance significantly affected target detect performance (*F*(3, 15) = 19.86, *p*<.001, *η_p_^2^* = .80; Fig. 4). Randomising colour and luminance impaired target detection performance, with better detection rates in predictable sequence compared to random colour, luminance and target alone conditions (*ps*<.05). However, randomising colour and luminance did not eliminate contour integration, with better detection rates in these two conditions compared to target alone (*ps*<.01). Detection rates were comparable when colour and luminance were randomised (*p* = .45). A significant condition×contrast interaction was further analysed (*F*(27, 135) = 4.68, *p*<.001, *η_p_^2^* = .48). The enhancement of the predictable sequence compared to all other sequences was most evident at low target contrasts 0.5–1.25% (*Fs*≥8.16, *ps*<.01), but not at 0.25% and higher contrasts (1.5–2.5%) (*ps*≥.05). In comparison to target alone, detection performance was better in predictable, random colour and random luminance sequences between contrasts 0.75–2.5% (*Fs*≥5.10, *ps*<.05) and was not significant at very low target contrasts (0.25–0.5%) (*Fs*≤3.01, *ps*≥.05).

The results suggest that favourable conditions for dynamic contour path integration occur when the contour elements (predictors) are of the same colour and luminance. This is consistent with the previous findings that we tend to group contour elements of the same colour [10]. Given that randomising colour and luminance did not show significant differences in target detection performance, our results support evidence which suggests that static contour integration for chromatic and luminance elements is similar [41] and this extends to dynamic global contour integration.

### Experiment 4: manipulation of predictor's orientation and alignment

Earlier investigations with static [2], [43] and dynamic [25] stimuli have shown that contour elements which share the same orientation are easier to integrate than elements which are randomly orientated, and elements which are aligned are easier to integrate than those that are misaligned [8]. Neurophysiological studies have also suggested that elements need to be precisely aligned for simple V1 cells to effectively integrate contour signals [44], [45]. However, there is little experimental work to directly compare the influence of orientation and alignment, particularly in dynamic contour integration.

#### Method

To examine the effects of orientation and alignment in contour path integration, the stimulus structure was manipulated in four sequences: (1) *Predictable sequence*; (2) *Random orientation sequence*: predictors with random orientation (0–180° in steps of 22.5°) appeared successively towards the fovea, followed by the target; (3) *Misalignment sequence*: five horizontal predictors with randomised vertical position (in the range of ±1° in relation to the target position) appeared successively towards the fovea, followed by the target; (4) *Target alone sequence*. It was predicted that if V1 orientation selective neurons play a key role in dynamic contour integration then disrupting continuity in orientation and alignment between the path elements should reduce contour path integration and therefore target detection. Five volunteers participated in this experiment.

#### Results and Discussion

A 4 (stimulus sequences)×10 (target contrast levels) ANOVA illustrated that disrupting the orientation and alignment of the predictor-sequence impaired target detection (*F*(3, 12) = 15.03, *p*<.001, *η_p_^2^* = .79; Fig. 5). Specifically, the target detection performance was better in the predictable sequence compared to all other conditions (*ps*<.05). The misalignment condition produced better detection rates than target alone condition (*p*<.05), whereas the random orientation condition did not (*p* = .23). However, target detection performance was not significantly different in random orientation and misalignment condition (*p* = .21), therefore it should only be tentatively suggested that randomising orientation impairs spatiotemporal contour integration greater than misalignment. A significant condition×contrast interaction (*F*(27, 108) = 4.71, *p*<.001, *η_p_^2^* = .54) demonstrated that between 0.5–1.75% target contrast performance was better in the predictable sequence compared to all other conditions (*Fs*≥7.25, *ps*<.01), but these effects were not significant between predictable sequence and random orientation or misalignment sequence at higher contrasts (2–2.5%) (*ps*>.05). At contrasts 0.75–1.75% target detection was better in misalignment compared to target alone condition (*Fs*≥9.66, *ps*<.01), but not at lower (0.25–0.5%) and higher (2–2.5%) contrasts (*p*>.05).

These results suggests that dynamic contour integration is better when the contour elements are spatially co-aligned and of the same orientation. However, reducing the spatial alignment between the predictor bars impaired target detection less than randomising predictor orientation, suggesting that orientation is more disruptive to contour integration than element alignment when modulated in the space-time domain. Given V1 neurons' sensitivity to orientation information [46], these results indicate that horizontal connections in area V1 could be heavily involved in dynamic contour path integration [6], [28]. This suggests that preferential connectivity between V1 columns may be more reliant on similar orientation preferences rather than direct alignment. Although apparent motion effects are typically reported in terms of shortening neural response latency [47], [48] it may contribute to our findings here. That is, the higher cortical areas could be involved in linking the contour elements based upon the motion trajectory [49], [50]. However, motion detectors are highly sensitive to spatial frequency [51] and the optimal temporal frequency for detecting coherent motion is between 59-24 Hz (17–42 ms) [52]. In experiment 1 and 2 we showed that dynamic contour path integration was relatively stable across different spatial gaps up to 2° and temporal gaps up to 200 ms. This suggests that although motion detectors are likely to play a role in processing the apparent motion produced by the stimuli, they are unlikely to be the primary contributors to dynamic contour path integration which biases target detection performance.

## General Discussion

The constraints on contour integration have typically been explored in static stimuli using a path detection task [2], [43], [53]. Given that we live in a dynamic visual world whereby visual inputs from different spatial and temporal windows are highly correlated [54], [55], it is beneficial that we also explore spatiotemporal contour integration to further define the parameters under which contour integration occurs. Overall, we observed that the human visual system effectively integrates spatially and temporally dispersed contour path information to facilitate target detection. These experimental findings are compatible with our previous psychophysical investigations [24], [25] which have suggested that we exploit prior knowledge of natural scene statistics (spatiotemporal regularity in this case) to facilitate the processing of current visual inputs [56]–[58]. For the rest of this discussion, we first compare our data to previously reported results, which have predominately used static stimuli and/or flanker-target-flanker designs, as opposed to the dynamic path integration used in our design. We then consider possible neural candidates for the basis of static and dynamic contour path computations.

Our experimental findings from the manipulation of spatial or temporal gap between predictors have suggested that dynamic collinear contour path integration was still evident when individual elements were separated by up to 2° spatial gap or 200 ms temporal gap (Fig. 2 and 3). This is relatively consistent with those observed in static contour integration, which suggest that collinear bar elements can be successfully integrated when the spatial gap does not exceed 2° [6]. Limited studies have reported the influence of temporal gap between contour elements, but in contrast to our findings reports suggest that flanker and target are only integrated when the target is presented within a 150 ms of the onset of the flanker [21]. Here we have demonstrated that integration of a dynamic contour path occurs over greater temporal gap than the integration of singular flanker-target stimuli, being robust at 200 ms intervals.

The present investigations are one of the first to directly compare the influence of orientation and alignment in dynamic contour integration tasks; this allows us to define more precisely the parameters of alignment which are important to successful contour integration [2], [59], [60]. In agreement with previous studies using static stimuli we have shown that integrating dynamic contour elements was more efficient (as evidenced by better target detection performance in Fig. 5) when the elements shared the same orientation and alignment [2], [8], [43]. We have also demonstrated that observers were potentially more sensitive to orientation cues than misalignment. Target detection performance was enhanced when the contour elements were of the same orientation but misaligned in comparison to when the elements were aligned but of different orientation. The reliance on orientation cues for successful contour integration is compatible with the functioning of V1 orientation-selective neurons [46]. Furthermore, dynamic contour path integration was better for contour elements defined by the same luminance contrast (predictable sequence in Fig. 4). This is comparable with static contour integration, which is more efficient for contours defined by achromatic elements [11]–[13]. Randomising elements' colour and luminance showed similar detrimental effect but did not abolish contour path integration, such that the observer could still integrate the elements to bias target detection performance. This suggests that the visual system is able to link similarly oriented dynamic contour elements even when luminance and colour cues are reduced.

These constraints on dynamic contour path integration are suggestive of the neural mechanisms underlying dynamic contour integration. Previous investigations using static stimuli have strongly suggested that contour elements can be integrated as early as area V1 through contextual interactions and intrinsic horizontal connections [2], [61]. For instance, V1 responses are facilitated by collinear line segments [6], [62] and closely correlate with the perceptual saliency of the static contours [15]. Recent extracellular recordings in rhesus monkeys have similarly reported the involvement of V1 neurons in the processing of dynamic contour path. Typically when the collinear predictors (extra-RF stimuli) and target (RF stimulus) were arranged as a dynamic predictable sequence, orientated towards and through to the neuron's RF, half of the recorded neurons responded to the predictors presented outside their RFs at the time that there was no visual stimulus presented inside the RFs [28]. However, findings from human ERPs [29], [30] and fMRI studies [63] have indicated the crucial role of later processing stages and neural generators beyond V1 in grouping dynamic contours. Our current findings provide original evidence to suggest that (as in primates) V1 is also critically involved in contour integration in the space-time as well as space domain.

To elaborate, the ability to integrate contour elements over a range of spatial (up to 2°) and temporal (up to 200 ms) spacing is harmonious with the characteristics of horizontal connections in V1 [6], [23]. Additionally, the importance of orientation information compared to alignment information is compatible with V1 orientation selective neurons communicating predictive information about the appearance of upcoming targets based up on their motion trajectory. Evidence from V1 neuronal populations shows that V1 responses increase in amplitude in the contour region (region of co-oriented contour elements) and decrease in the background region (region of randomly oriented elements), indicating that V1 is actively involved in the perceptual grouping of similarly oriented elements [61]. Computational models have suggested that recurrent excitatory and inhibitory horizontal connections in V1 could sub-serve this process, prioritising targeting cells which are linked with similar orientation preferences [64].

It is also thought that V1 orientation selective neurons show limited selectivity to chromaticity [40], [42]. The performance data in Fig. 4 fits well with this neurophysiological observation. The target detection was better when the grey contour elements were of the same luminance contrast, yet randomising colour and luminance did not eliminate integration altogether, with better performance in the random colour and luminance condition compared to target alone. This illustrates that V1 neurons may be communicating information to predict the target based upon linking of contour elements. The strength of these connections is likely to be stronger when connecting neural responses which share similar orientation, colour and luminance preferences. When colour and/or luminance preferences are not matched the strength of the connections are reduced, such that target detection was decreased, but not eliminated, such that target detection was better than when the target is presented without the contour information (target alone condition). This speculation is supported by the earlier neurophysiological observation that a population of V1 neurons (∼30%) showed approximately equal orientation selectivity to both chromatic and luminance gratings [65], [66] suggesting horizontal connections in V1 can still function with color-defined orientated colour elements.

On the other hand, the integration of coherent but spatially and temporally separated visual signals is often subject to the influence of top-down modulation (e.g., expectation and prediction), and has traditionally been ascribed to the neural processes in higher cortical areas, such as frontal and parietal cortex [26], [67], [68]. Indeed, some evidence suggests that contour integration responses in monkey V1 is absent when the task is novel and when under anaesthesia (passive viewing). With active perceptual learning V1 shows delayed contour integration responses, which are thought to be the result of recurrent top-down processes [69]. Furthermore, recordings of ERPs showed similar contour integration processes in humans with dynamic sequences [30]. When dynamic contour paths were passively viewed (no task), or when attention was taken away from the path (by a secondary colour counting task), the shortened N1 peak latency associated with viewing targets embedded in dynamic predictor-target paths was abolished. Because N1 peaks at relatively later stages of processing (i.e. post 200 ms), it was suggested that this component reflects the involvement of top-down processes, and these processes are imperative to successfully linking contour elements to bias target detection [30]. However, a recent ERP study has shown that even at very early stages of processing, at a time window associated with V1 processing (∼66 ms), contour integration of predictable path elements (co-linear paths) shortens peak latencies of early ERP components in comparison to the integration of less predictable paths (co-circular paths) [31]. This suggests that V1 may play an independent role in human contour integration. Although we cannot differentiate the relative contribution of long-range horizontal connections and feedback connections in dynamic contour integration in this study, the similar constraints on the integration of static and dynamic contour path suggests that V1 neurons are directly involved in the dynamic contour integration.

In conclusion, using dynamic contour elements the investigations reported here illustrate that human observers utilise contour path information modulated in space and time to facilitate their detection of low contrast targets. The spatial, temporal, colour/luminance and alignment parameters under which this performance contribute to the growing debate within the literature as to the parameters under which contour integration is facilitated and broadly supports with the functioning of V1 neural processes. It seems that the visual system can integrate dynamic contour paths to bias target detection even when the path is disrupted by spatial and temporal intervals and breaks in alignment, colour and luminance.
